# Metagenomics of plant and fungal viruses reveals an abundance of persistent lifestyles

**DOI:** 10.3389/fmicb.2014.00767

**Published:** 2015-01-12

**Authors:** Marilyn J. Roossinck

**Affiliations:** Department of Plant Pathology and Environmental Microbiology, Center for Infectious Disease Dynamics, Pennsylvania State UniversityUniversity Park, PA, USA

**Keywords:** virus ecology, mycoviruses, dsRNA viruses, persistent plant viruses, mutualistic viruses

## Introduction

Most studies of plant viruses have focused on the acute viruses that cause disease in crop and ornamental plants. These viruses are transmitted horizontally, often by insect vectors, and are occasionally transmitted vertically. Although known for at least four decades, the persistent viruses of plants are very poorly studied. These viruses were previously called “cryptic” because they did not appear to illicit any symptoms in infected plants (Boccardo et al., 1987). Persistent plant viruses are not known to be transmitted horizontally, although phylogenetic evidence suggests some level of transmission (Roossinck, 2010). They are vertically transmitted at nearly 100% levels through both ova and pollen (Valverde and Gutierrez, 2007). They have been identified in metagenomic studies by their similarity to known persistent viruses, and because they lack any movement protein, a feature of all known acute viruses that must move through the plant plasmodesmata to establish a systemic infection. Persistent viruses do not move between plant cells, but rather infect every cell and move by cell division.

Most plant persistent viruses have double-stranded (ds) RNA genomes, and encode only an RNA dependent RNA polymerase (RdRp) and a coat protein. Of the well-characterized persistent plant viruses, those in the *Endornaviridae* are the exception. These viruses have a single-stranded (ss) RNA genome, based on their RdRp, and encode a large polyprotein that does not have any apparent coat protein, but encodes a number of additional domains that appear to be derived from diverse sources (Roossinck et al., 2011). They are usually found as dsRNA replicative intermediates.

Viruses of fungi have very similar lifestyles to plant persistent viruses, and several virus families are shared between plants and fungi. Phylogenetic evidence indicates that virus transmission has occurred within and between the two kingdoms (Roossinck, 2010; Roossinck et al., 2011).

Fungal viruses are even less well-studied than plant viruses, and the diversity of these viruses remains mostly unknown. A majority of known fungal viruses have dsRNA genomes, some have ssRNA genomes, and a few examples of DNA viruses are known (Yu et al., 2010). Recently a negative sense ssRNA virus was characterized from a fungus (Liu et al., 2014). Similar to plant viruses, most fungal viruses have been studied in the context of pathogenic fungi. The discovery of the hypovirulence phenotype of *Cryphonectria hypovirus* 1 that suppresses the disease phenotype of the chestnut blight fungus led to a search for other examples that could be exploited to mitigate the effects of plant pathogenic fungi [reviewed in Dawe and Nuss (2013)].

## Virus discovery in plants and fungi

Deep sequencing is proving to be a useful technique for just about everything these days, and the methods have been applied to metagenomic studies of viruses. Unlike studies of other microbes, viruses cannot be analyzed through the use of any universal conserved sequences or motifs, and a variety of techniques have been employed to enrich for viral nucleic acids before sequence analysis. Studies in aquatic viruses have been reported for a number of years (Angly et al., 2006; Labonté and Suttle, 2013). More recently plant viruses have been studied through metagenomics as well (Roossinck, 2012; Stobbe and Roossinck, 2014). A large variety of studies have been done on many different scales, from individual plants to ecosystems. In some studies a single plant species has been targeted, in others a broader sweep is used. These studies are explored in detail in a review by this author and others to be published elsewhere. Here I will explore the discovery of persistent viruses that are extremely common in plants and fungi, but poorly studied, and discuss the implications of these viruses in the ecology of plants and fungi.

Different methods of detection have yielded different levels of persistent viruses. Use of dsRNA-enriched samples yielded very high levels of persistent viruses in plants (Roossinck, 2012). Using the small RNAs involved in plant immunity (siRNAs) has been less successful at detecting many persistent viruses in plants. While the complete sequence of a known endornavirus was assembled with siRNAs (Sela et al., 2012), no novel endornaviruses have been reported with this method. A few siRNAs have been found for partitiviruses, chrysoviruses, and totiviruses, but with very limited genome coverage (Kreuze, 2014). It is likely that these viruses are not subjected to silencing; with the exception of endornaviruses, they do not expose their dsRNA to the cell, but rather retain their genomes within the virions and extrude only ssRNA into the cytoplasm (Safari and Roossinck, 2014).

Virus discovery in fungi is very limited. Most analyses have been done on fungi of economic importance such as plant pathogenic fungi. A survey of viruses from endophytic fungi derived from two plant species in a wild plant community indicated that the diversity of viruses in this system was much greater than the diversity of fungi, which was in turn much greater than the diversity of plants (Feldman et al., 2012).

In most cases of fungal virus studies, viruses have been discovered from cultured fungi. This eliminates the majority of fungi, which are not culturable (Blackwell, 2011), but have been discovered from environmental samples through specific gene analysis such as ribosomal RNA-related regions and other genes (Seifert, 2009). Traditionally fungi acquired from nature are “purified” by single spore isolation. These practices result in a gross under-estimate of fungal viruses, as many viruses are lost during culture, especially on solid media (unpublished observation), and single spore isolation is a common strategy to obtain cultures “cured” of their viruses. Even though next generation sequencing methods allow for deeper analysis of environmental samples, finding new viruses in fungi without culture is technically challenging. Although some reports have indicated that fungal viruses can be shed into the media when cultured, there is little evidence of extracellular accumulation of most fungal viruses. The lack of any conserved sequences in viruses, such as house-keeping or bar-coding genes found in all other life forms, means that sequence-specific primers cannot be used. For viruses of endophytic fungi that have been the focus of fungal virus research in the author's lab, the minimal amount of fungal tissue in plants makes any analysis nearly impossible without culturing the fungus out of the plant. Hence for now we must settle for this very low estimate of fungal virus diversity.

## Common themes from plants and fungi

Plants and fungi share several families of viruses. The *Partitviridae* and the *Endornaviridae* are recognized by the International Committee for the Taxonomy of Viruses (King et al., 2012) as infecting both plants and fungi, but biodiversity surveys of plant viruses have also identified members of the *Totiviridae* and *Chrysoviridae* families that traditionally are considered fungal viruses (Roossinck, 2012), and a chrysovirus was recently characterized from radish (Li et al., 2013). In fact viruses from these and related families make up over half of the viruses identified in wild plants (Roossinck, 2012). In plants these viruses appear to maintain a persistent lifestyle (Roossinck, 2010), remaining associated with their hosts for many generations with nearly 100% vertical transmission. Less is known about the lifestyles of fungal viruses. There are few reports of truly acute viruses in fungi. Recently a DNA virus from *Sclerotinia sclerotiorum* was shown to be infectious as a purified virus particle, although it is not clear if this is a mechanism for transmission in nature (Yu et al., 2013). Unlike the plant persistent viruses, fungal viruses can be transmitted between closely related strains of fungus through anastomosis (Milgroom and Hillman, 2011), and evidence of cross-species transmission is apparent in phylogenetic analyses of *Cryphonectria hypovirus* (Liu et al., 2003) and partitiviruses in the *Heterobasdion* (Vainio et al., 2011).

Persistence and high levels of vertical transmission in parasites are correlated with commensal or mutualistic lifestyles (Villarreal, 2007; Márquez and Roossinck, 2012). In some cases we know that persistent viruses are mutualistic (Nakatsukasa-Akune et al., 2005; Márquez et al., 2007). In many cases we don't know enough about their biology to assess their symbiotic lifestyle, but in plants few have any evidence of negative effects on their hosts. For two persistent viruses in *Heterobasidion* species, virus lifestyle was dependent on the fungal environment (Hyder et al., 2013). A complicating factor in understanding the ecology of persistent viruses is that the well-studied persistent viruses are mainly from crop plants, or from economically important fungi; there is virtually no information about any roles these viruses may play in the natural environment where the virus-host relationships evolved.

## Conclusions

The abundance of persistent viruses in plants and fungi imply functions that may contribute to the biology of the host. Unfortunately we have little ecological data about these viruses, and since they often cause no disease they have not been the subject of intensive study. Data-mining from transcriptomic, genomic and metagenomic studies may allow us to address the true ecological role of these viruses. For example, the partitiviruses have poly-A tails, and may be detectable in transcriptome analyses (Jiang et al., 2013). In some cases persistent virus sequences are found integrated into plant or fungal genomes (Liu et al., 2010; Chiba et al., 2011). Deeper analyses along these lines may provide data on time-lines of persistent virus-host relationships.

### Conflict of interest statement

The author declares that the research was conducted in the absence of any commercial or financial relationships that could be construed as a potential conflict of interest.
